# A Panel of MicroRNA Signature as a Tool for Predicting Survival of Patients with Urothelial Carcinoma of the Bladder

**DOI:** 10.1155/2018/5468672

**Published:** 2018-06-20

**Authors:** Teruo Inamoto, Hirofumi Uehara, Yukihiro Akao, Naokazu Ibuki, Kazumasa Komura, Kiyoshi Takahara, Tomoaki Takai, Taizo Uchimoto, Kenkichi Saito, Naoki Tanda, Yuki Yoshikawa, Koichiro Minami, Hajime Hirano, Hayahito Nomi, Ryuji Kato, Tetsuya Hayashi, Haruhito Azuma

**Affiliations:** ^1^Department of Urology, Osaka Medical College, Takatsuki, Osaka, Japan; ^2^United Graduate School of Drug Discovery and Medical Information Sciences, Gifu University, Gifu, Japan; ^3^Cardiovascular Pharmacotherapy and Toxicology, Osaka University of Pharmaceutical Sciences, Takatsuki, Japan

## Abstract

**Introduction and Objectives:**

MicroRNA (miRNA) expression is altered in urologic malignancies, including urothelial carcinoma of the bladder (UCB). Individual miRNAs have been shown to modulate multiple signaling pathways that contribute to BC. To identify a panel of miRNA signature that can predict aggressive phenotype from normal nonaggressive counterpart using miRNA expression levels and to assess the prognostic value of this specific miRNA markers in patients with UCB.

**Methods:**

To determine candidate miRNAs as prognostic biomarkers for dividing aggressive type of UCB, miRNA expression was profiled in patients' samples with an aggressive phenotype or nonaggressive phenotype using 3D-Gene miRNA labeling kit (Toray, Japan). To create a prognostic index model, we used the panel of 9-miRNA signature based on Cancer Genome Atlas (TCGA) data portal (TCGA Data Portal (https://tcgadata.nci.nih.gov/tcga/tcgaHome2.jsp)). miRNA expression data and corresponding clinical data, including outcome and staging information of 84 UCB patients, were obtained. The Kaplan-Meier and log-rank test were performed to quantify the survival functions in two groups.

**Results:**

Deregulation of nine miRNAs (hsa-miR-99a-5p, hsa-miR-100-5p, hsa-miR-125b-5p, hsa-miR-145-5p, hsa-miR-4324, hsa-miR-34b-5p, hsa-miR-29c-3p, hsa-miR-135a-3p, and hsa-miR-33b-3p) was determined in UCB patients with aggressive phenotype compared with nonaggressive subject. To validate the prognostic power of the nine-signature miRNAs using the TCGA dataset of bladder cancer, the survival status and tumor miRNA expression of all 84 TCGA UCB patients were ranked according to the prognostic score values. Of nine miRNAs, six were associated with high risk (hsa-miR-99a-5p, hsa-miR-100-5p, hsa-miR-125b-5p, hsa-miR-4324, hsa-miR-34b-5p, and hsa-miR-135a-3p) and three were shown to be protective (hsa-miR-145-5p, hsa-miR-29c-3p, and hsa-miR-33b-3p). Patients with the high-risk miRNA signature exhibited poorer OS than patients expressing the low-risk miRNA profile (HR = 7.05, *p* < 0.001).

**Conclusions:**

The miRNA array identified nine dysregulated miRNAs from clinical samples. This panel of nine-miRNA signature provides predictive and prognostic value of patients with UCB.

## 1. Introduction

Progress has been made in the last two decades in understanding the complex genetic dysregulation in UCB, yet the attempt to pursue an effective treatment of metastatic or advanced UCB seems to be futile. The treatment of patients with locally advanced UCB is challenging because of high recurrence rate after radical cystectomy (RC). Standard of cure for muscle invasive bladder cancer (MIBC) is RC with lymphadenectomy [1]. However, the durable cure rate after RC is actually smaller than the cure rate of other solid genitourinary malignancies leading urologists to attempt to improve long-term outcomes [1]. The current therapeutic options for metastatic UCB are particularly limited except for the latest immunological checkpoint inhibitors because of significant number of resistance rate to chemotherapy; there is a need to analyze the genetic mechanisms in order to determine specific targets for precision therapy. With the development of computer-based assessment tools for miRNA detection and biochemical prediction to combine varying miRNAs in vitro and in vivo model, novel tools of the miRNA field are growing. miRNA is a small noncoding RNAs that regulate a wide range of functional properties through changing the translation accompanied with expression of their target mRNAs. Accumulating facts have demonstrated that altered expression of miRNAs in human malignancies results in the deregulation of the levels of oncogenes and antioncogenes, which promotes the proliferation of cancers. Therefore, miRNA-based detection tools for the purpose of precision medicine emerged as an effective option and may offer a curative potential in UCB therapy, either alone or in combination with other therapies. Due to their well-established selectivity and specificity, miRNAs can represent a useful tool, both in diagnosis and therapy, in forging the path towards the achievement of precision medicine [2]. The aim of the present study was to focus on the possible role of miRNA dysregulation in UCB and discuss the potential of miRNAs as prognostic biomarkers.

## 2. Methods

### 2.1. Validation of the Expression Profiles of miRs in Quantitative Real-Time Polymerase Chain Reaction (qRT-PCR)

Three Japanese patients with advanced MIBC were enrolled in the present study. Patient 1 was a 73-year-old male patient with the tumor being diagnosed as T2 stage (control); patient 2 was an 85-year-old male patient—the tumor was T3 stage (nonaggressive phenotype); patient 3 was a 67-years-old male patient—the tumor was T2 stage as well (aggressive phenotype). The histopathology of the specimens was determined by transurethral resection of the tumor. The tumor histologic grade and type of these three patients were high grade urothelial carcinoma. On the basis of the American Joint Committee on Cancer (AJCC) TNM staging system, the tumor stages of patients 1–3 were stage II (cT2N0M0), stage III (cT3N0M0), and stage III (cT2N0M0). The tumor morphology of all three patients was sessile and multiple. Before the samples were taken, none of these three patients underwent chemotherapy and radiotherapy. Although sex and chronological age differed among patients included in the first analysis, we included patients who were treated with the same treatment protocols, so that we could minimize the effect of heterogeneity of treatments. All three patients underwent curative treatment. Patient 1 and patient 2 were cured with no evidence of disease. Three months after the therapy, patient 3 developed multiple bone metastases, massive edema of the lower limbs, and peritoneal ascites with progressive worsening of the blood sampling. The patient died seven months after the treatment.

The informed consents of all the participants were obtained. Bladder specimens were immediately stored in the tissue in the nitrogen after the resection. Whole RNA was extracted from the tissue by the 3D-Gene® RNA extraction reagent (Toray, Tokyo, Japan). Upon extraction from the tissue sample, the tissue was sliced into small pieces smaller than 5 mm. RNA was labeled and hybridised on the chip with 3D-Gene miRNA labeling kit to detect >2500 types of miRNAs [3–6]. Normalized data processing was conducted thereafter. The microarray was scanned, and the images were obtained. To generate raw data, 3D-GeneH scanner 3000 (Toray, Tokyo, Japan) was used to extract the fluorescent signals from images. The expression status of miRNAs was normalized by the removal of mean background signal intensity from the entire set of miRNAs in each microarray, which is generally called as globally normalization method, with threshold for detected signal > the mean + 2x SD of the blank spot signals.

Previous studies have suggested that the altered expression of miRs was associated with poor prognosis of UCB. To synthesize multiple microarray-based human UCB miRNA expression profiling, we employed a vote-counting strategy to identify several consistent differentially expressed miRNAs, of which rank potential molecular markers widely adopted in the meta-analysis [7, 8]. We ranked miRs according to the importance of each miR. We considered the number of consistent comparisons reported previously, total number of samples in agreement, and average fold changes reported for comparisons in agreement. For three of these, total sample size was deemed as the most important factor than the average fold change.

### 2.2. Validation of the Expression Profiles of miRs in The Cancer Genome Atlas (TCGA) Database

To assess multivariable prognostic miRNA expression profile as biomarkers and to evaluate in several cohorts, curated and updated tools of miRNA expression levels associated with outcome evaluation that provides survival analysis established by Aguirre-Gamboa and Trevino were utilized [9]. The percentages of tumor stage of 84 UCB patients were stage I (1.0%), stage II (22.0%), stage III (39.0%), and stage IV (38.0%). As depicted by Aguirre-Gamboa and Trevino, patient data were searched for keywords related to malignancies, survival information, and miRNA signatures [9]. Cohort searches are based on four large databases which are GEO (http://www.ncbi.nlm.nih.gov/geo), GEOmetadb [10], ArrayExpress (https://www.ebi.ac.uk/arrayexpress/), and level 3 TCGA (https://tcga-data.nci.nih.gov/tcga) [9]. In this platform, RNAseq generates TCGA read counts and transformed in to log2 [9].

## 3. Results

**Figure 1:**
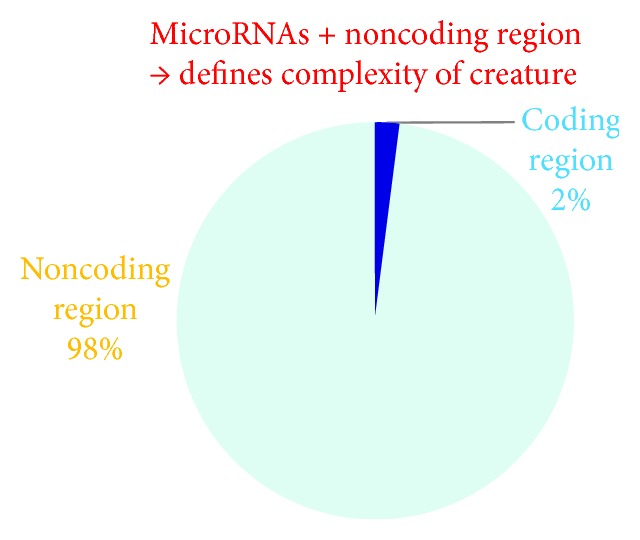
Putative concept showing that higher orgasms contains more noncoding region.

As suggested by Dr. Taft and Dr. Mattick from Australia, the ratio of noncoding to total genomic DNA for numbers of sequenced species correlates with increasing biological complexity [11, 12]. They suggest that the observed noncoding DNA increases and compositional patterns are primarily a function of increased information content [11, 12]. It is conceivable that introns and genomic DNA previously regarded as genetically unnecessary may be more meaningful. Approximately 2% of the mammalian genome encodes miRs in exons or introns of protein-coding genes or intergenic regions, and miRs may be clustered or found in isolation [13]. Perhaps, miRs coming mostly from noncoding region that defines complexity of creature are important (Figure 1).

**Figure 2:**
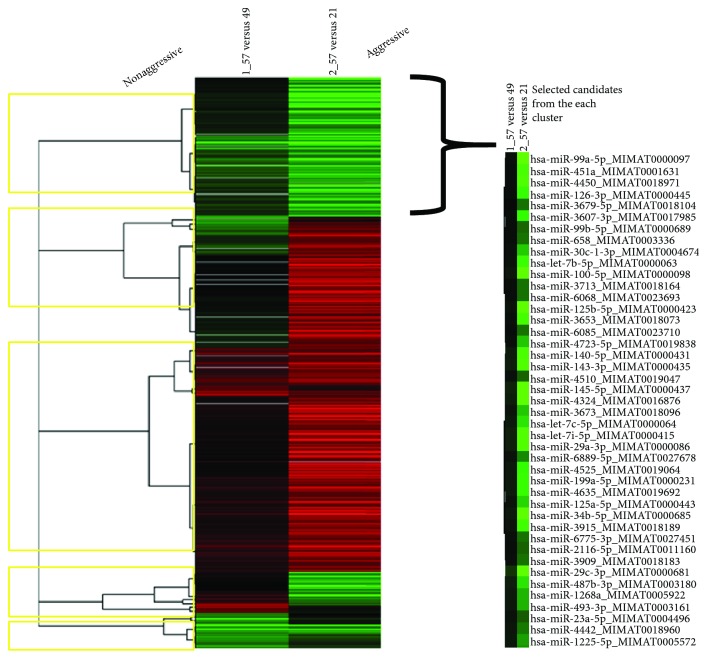
Clustering and heat maps by two different UCB phenotypes. Red signal shows the amount of gene expression activity, and green signal measures the gene expression activity for a control.

**Figure 3:**
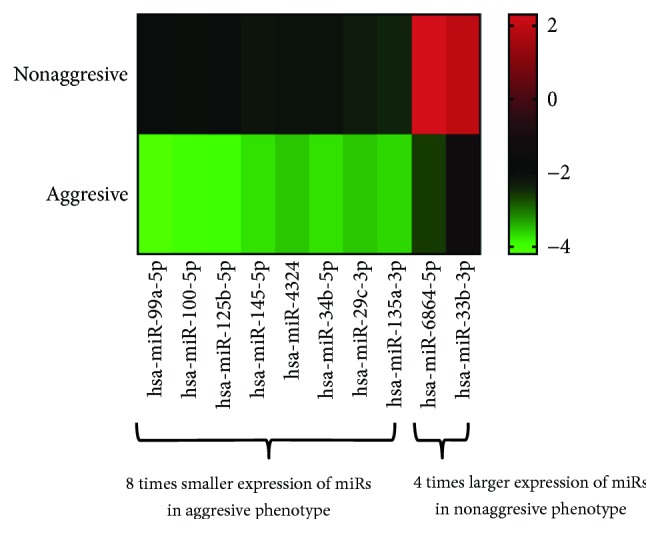
Heat maps by two different UCB phenotypes. Variable ratio < 8 times or >4 times had 8 and 2 genes, respectively.

**Figure 4:**
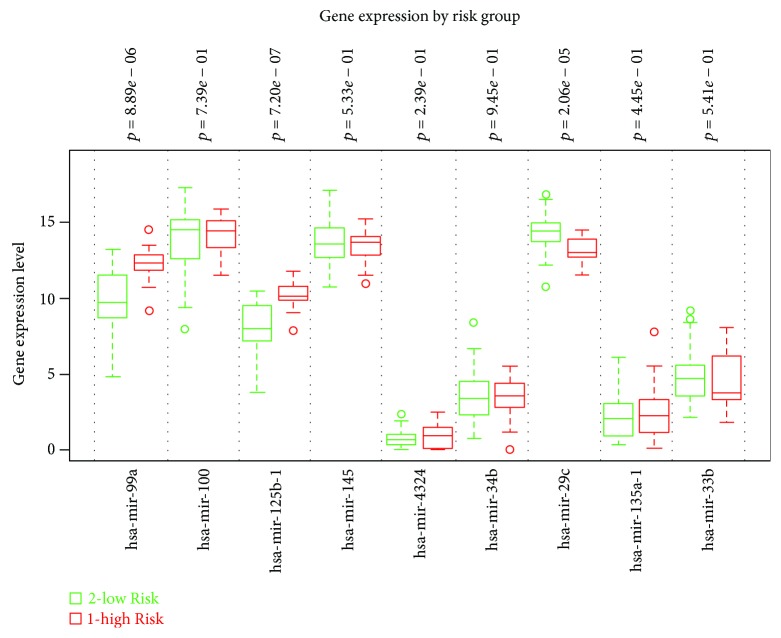
Gene expression by risk group. The expressions of genes with low risk (green) and high risk (red) are indicated.

**Figure 5:**
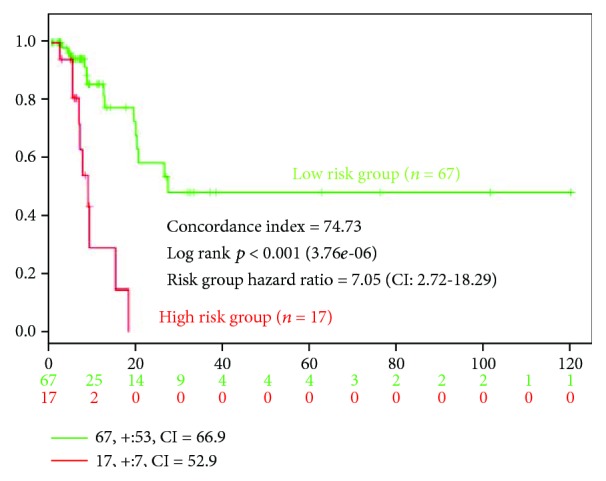
Kaplan-Meier survival curves stratified by three different subgroups. The survival expressions in patients with low risk (green) and high risk (red) are indicated.

To find a panel of miRNAs as biomarker for UCB, the present study initiated with the selection of specific miRNA candidates based on the comparison of expression levels in cancerous tissue between UCB patients with aggressive phenotype and nonaggressive phenotype adjusted by representative UCB by means of the Toray 3D-Gene miRNA array. During the selection of candidate UCB miRNA for miRNA panel from the comprehensive miRNA array-based approach, we selected candidate miRNAs for aggressive phenotype detection based on comparison of the expression levels of each miRNA between UCB patients with aggressive phenotype and nonaggressive counterpart (Figure 2). To implement a clustering algorithm, Pearson correlation was used to measure similarity (Figure 2). Correlation coefficient was calculated between the intensities measured for each miR and the values of the independent variable. Fold change was used in analysis of expression data in microarray and was calculated as the ratio of the difference. Value was the log 2 transformation of the normalized ratio of the average red signal and the average green signal, and 602 genes that have 2 times larger log 2 of red/green normalized ratio were used. In order to find more sensitive biomarkers, we focused on 10 miRs with the highest alteration levels in the tissue of UCB patients (Figure 3). Of these 10 miRNAs, we chose 9 miRNAs, hsa-miR-99a-5p, hsa-miR-100-5p, hsa-miR-125b-5p, hsa-miR-145-5p, hsa-miR-4324, hsa-miR-34b-5p, hsa-miR-29c-3p, hsa-miR-135a-3p, and hsa-miR-33b-3p which were previously reported to have an oncogenic or antioncogenic roles [5, 14–21], (Figure 4). Our previous studies found that the expression levels of antitumorigenic miR-145 were significantly lower in surgically resected BC samples and BC cell lines compared to those in normal bladder tissues, thereby miR-145 decreases the Warburg effect by silencing KLF4 [22, 23]. Concerning the combination of oncogenic and/or antioncogenic miRNAs, the prognostic role of the specific expression profile of miRNAs is currently uncertain in BC, because most of the previous studies focused on oncogenic miRNAs or vice versa [24–27]. In this study, therefore, we conducted combined profiles of 9 miRNAs for further analyses, because these miRNAs need considerable specific investigation at the present. To determine if the 9 most deregulated miRNAs from the analysis predict CSS in UCB, we performed the analysis of metasignature of miRNAs as predictive biomarkers for UCB. Our result showed that upregulated miR-99a and miR-125b levels and downregulated miR-29c levels were associated with high risk feature (8.89 × 10^–6^, 7.20 × 10^−7^, and 2.06 × 10^−5^; Figure 4). There is no significant association between other dysregulated miRNAs (miR-100, miR-145, miR-4324, miR-34b, miR-135a, and miR-33b) (Figure 4; all *p* > 0.05). In the cohort, we conducted univariate analysis, age ranged from 43 through 86. In total, 60 events occurred during the follow-up. Patient survival curves were created based on poor outcome risk from TCGA data using the SurvMicro database. CI of the combined signature was 0.74 (Figure 5).

## 4. Discussion

The present study shows that the specific signature of miRs is associated with a higher rate of cancer-related deaths in patients with UCB. For locally advanced UCB, RC with or without platinum-based chemotherapy is still the only definitive treatment, but after the curative treatment, significant portion of patients experienced recurrence. Despite the likelihood for successful surgical excision of the tumor-invading bladder and the potential of curative treatment, the long-term outcome and mortality of MIBC are not easy to accurately predict. The 10 y overall survival, cancer-specific survival, and recurrence-free survival rates of MIBC managed by surgery are reported to 44%, 67%, and 66%, respectively [28]. Searching for survival biomarkers that enable the precise outcome for patients with MIBC has emerging as combining basic and clinical cancer research. MicroRNAs are currently discovered as a class of short noncoding RNA, which is known to regulate posttranscriptional gene expression level, by binding to homologically identical sequences, to the 3′-UTR of target miRNAs, thereby resulting in translational inhibition accompanied with miRNA degradation [29]. The expressions for miR-99a or miR-125b were downregulated in aggressive tumor (Figure 3) but altered when combined with other miRs (Figure 4). To maximize the concordance index, we have used algorithm to find the specific sets of microRNA expression signature that belong to different roles. In combination with other miRs, it is conceivable that miR-99a or miR-125b dynamically showed altered expression status relatively affected by expression status by other miRs. We have used miR-99a, miR-100, miR-125b, miR-145, miR-4324, miR-34b, miR-29c, miR-135a, and miR-33b for stratifying patients with aggressive and nonaggressive type of UCB. Guancial et al. reviewed the literature on the role of miRs in UCB and summarized that NMIBC demonstrates downregulation of miR-99a, miR-100, miR-101, and miR-145 compared with MIBC [30]. MiR-99a targets fibroblast growth factor receptor (FGFR), of which mutations and/or overexpressions are validated to be common in UCB [31]. miR-29 is known to play a role in invasive ability in UCB [32]. The ataxia-telangiectasia group D complementing (ATDC) gene, an oncogene, was found to suppress miR-29, leading to DNA methylation and silencing of the tumor suppressor PTEN [32]. The diagnostic values of these miRNAs in varying tumors are reported [5, 14–21]. Among all, miR-125b especially in biofluids appears to be so far more investigated than other miRNAs evaluated in the present study. According to the systematic review by Regazzo et al., miR-125 in gliomas plays a role as an oncogene and also is downregulated in cancer stem cells to have an antiangiogenic role, concluding that deregulation is different in varying types of tumor, due to the dual role of this miRNA as oncogenic miRNAs or tumor suppressor depending on tissue type or context [33]. This is valid also in the case of UBC where the direction of miRNAs varies from other malignancy, underlining the importance of profiling miRNAs specific on UCB.

## 5. Conclusions

Although survival biomarkers for UCB are leveraged in the clinical settings to classify specific cancer types, no such standard biomarkers have been identified in patients with UCB. The results of the present study highlight the aggressive type of UCB by means of array of miRNA profile. The present panel of miRNA profile will aid to the individual management of UCB.
